# Soil water capture trends over 50 years of single-cross maize (*Zea mays* L.) breeding in the US corn-belt

**DOI:** 10.1093/jxb/erv430

**Published:** 2015-10-01

**Authors:** Andres Reyes, Carlos D. Messina, Graeme L. Hammer, Lu Liu, Erik van Oosterom, Renee Lafitte, Mark Cooper

**Affiliations:** ^1^DuPont Pioneer, 18369 County Rd 96, Woodland, CA, USA; ^2^DuPont Pioneer, 7200 NW Avenue, Johnston, IA 50310, USA; ^3^Queensland Alliance for Agriculture and Food Innovation, The University of Queensland, Brisbane, Queensland 4072, Australia

**Keywords:** Grain yield, maize, plant breeding, rooting, soil water uptake.

## Abstract

Breeders have successfully improved maize (*Zea mays* L.) grain yield for the conditions of the US corn-belt over the past 80 years, with the past 50 years utilizing single-cross hybrids. Long-term improvement for grain yield under water-limited conditions has also been reported. Grain yield under water-limited conditions depends on water use, water use efficiency, and harvest index. It has been hypothesized that long-term genetic gain for yield could be due, in part, to increased water capture from the soil. This hypothesis was tested using a set of elite single-cross hybrids that were released by DuPont Pioneer between 1963 and 2009. Eighteen hybrids were grown in the field during 2010 and 2011 growing seasons at Woodland, CA, USA. Crops grew predominantly on stored soil water and drought stress increased as the season progressed. Soil water content was measured to 300cm depth throughout the growing season. Significant water extraction occurred to a depth of 240–300cm and seasonal water use was calculated from the change in soil water over this rooting zone. Grain yield increased significantly with year of commercialization, but no such trend was observed for total water extraction. Therefore, the measured genetic gain for yield for the period represented by this set of hybrids must be related to either increased efficiency of water use or increased carbon partitioning to the grain, rather than increased soil water uptake.

## Highlight

Soil water capture potential of maize has not changed over 50 years of single-cross breeding. Changes in resource use efficiency and allocation to reproductive organs must underpin yield improvement.

## Introduction

Maize yield in the US corn-belt has increased steadily for over 80+ years and doubled between 1965 and 2010 (Smith *et al.*, 2014). Improvements in germplasm, agronomic practices, and cropping systems intensification contributed to these sustained yield gains. The long-term genetic gain in maize yield for the conditions of the US corn-belt over the past 80 years was demonstrated by quantifying the productivity of successful hybrids commercialized over eight decades; these hybrids are known as the ERA hybrids (Duvick *et al.*, 2004; Duvick, 2005; Campos *et al.*, 2006; Cooper *et al.*, 2014). Results from these ERA studies highlighted the dependency of yield genetic gain on plant population, reduction of the interval between anthesis and silking (anthesis-silking interval, ASI), decrease in the number of plants that do not set ears under high plant densities, and limited change in harvest index (HI) (Duvick *et al.*, 2004). When these results are considered together and analysed in the context of the negative association between grain yield and ASI (Campos *et al.*, 2004)—which is an indicator of increased ear growth rate and resource availability per ovule (Edmeades *et al.*, 1993)—they lead to the hypothesis that genetic gain was determined by an increased stress tolerance and resource allocation to reproductive organs (Duvick, 2005). This hypothesis was evaluated with tropical germplasm selected for improved drought tolerance (Bolaños and Edmeades, 1993; Edmeades *et al.*, 1999), and Argentinian flint and semi-dent hybrids (Echarte *et al.*, 2000; Luque *et al.*, 2006), but still remains to be evaluated for US temperate maize.

The correlated response of genetic gain for yield in the USA between well-watered and drought environments, and the selection in a target population of environments where biotic and abiotic challenges including drought are frequent, suggest that multiple physiological mechanisms contributed to the observed genetic improvement of yield (Duvick, 2005; Cooper *et al.*, 2014). It is plausible that the reduction in ASI and the apparent increase in carbohydrate allocation to reproductive organs could result at least in part from improved plant, kernel, and ovule water status. The reduction in ASI as determined by early silk emergence rather than late shedding (Welcker *et al.*, 2007), the high sensitivity of silks to water deficit (Westgate and Boyer, 1985), the high correlation between silk emergence and kernel set (Schussler and Westgate, 1994), and the absence of changes with selection in total biomass in tropical germplasm (Edmeades *et al.*, 1999), provide evidence to support this interpretation. Results from a simulation study, conducted to consider the roles of leaf and root architecture in light and water capture, helped formulate the hypothesis that, changes in root architecture associated with improved soil occupancy and water capture could be a process underpinning the observed hybrid by density interactions in historical maize yield improvement in the USA (Hammer *et al.*, 2009). The onset of accelerated yield improvement under drought stress conditions that coincides with the initiation of single-cross (SX) hybrids breeding in the 1960s (Cooper *et al.*, 2006), changes in root architecture (York *et al.*, 2015), and reduction in canopy temperature (Barker *et al.*, 2005) with the year of commercialization, and the contrast between water uptake patterns between single and double-cross hybrids (Campos *et al.*, 2004), supports the hypothesis that the observed trend in yield during SX breeding could be associated, at least in part, with an increased water capture.

The objective of this study was to test the hypothesis that water capture increased as the result of selection for yield in SX hybrids in the US corn-belt and that this process underpinned at least in part the genetic improvement in grain yield under drought stress. The results from this study can inform the design of breeding strategies that are aimed at developing germplasm that fully utilizes available water resources.

## Materials and methods

### Field experiments

Two field experiments were conducted in 2010 and 2011 at a DuPont Pioneer experiment station located 6 km west of Woodland, CA, USA. Eighteen ERA hybrids commercialized between 1963 and 2009 (Table 1) were planted in a Yolo Silt Loam (Fine-silty, mixed, superactive, nonacid, thermic Mollic Xerofluvents) (Andrews, 1972). The hybrids included in this study represented the SX hybrids that have previously been included in long-term genetic gain experiments for maize in the US corn-belt (Duvick *et al.*, 2004; Smith *et al.*, 2014). They represent a historical sequence of hybrids that were widely adopted by growers following their commercial release. Experimental evaluations under a range of water-limited environments, through use of managed drought environments at low rainfall locations, have demonstrated that this set of hybrids demonstrated genetic gain for both water-limited environments and high-input environments (Cooper *et al.*, 2014).

**Table 1.** Single-cross maize hybrids included in experiments conducted in 2010 and 2011 and their year of commercial release Soil water use was measured on all hybrids, except as indicated.

**Hybrid**	**Year**	**Hybrid**	**Year**
3306	1963	3489^a^	1994
3334	1969	3335^a^	1995
3366^c^	1972	33P66^b^	1999
3541^b^	1975	34G81^c^	1997
3377	1982	34H31^b^	2002
3475	1984	33D11^a^	2005
3379^a^	1988	33D49	2008
3394	1991	35F40	2007
3378 ^c^	1983	33W82^c^	2009

^a^ soil water measured only in 2010.

^b^ soil water measured only in 2011.

^c^ soil water was not measured in this hybrid.

The experiments were machine planted in two-row plots of 4.5 m long with 0.76 m spacing between rows. Plots within planting rows were separated by a 0.60 m alley and between planting rows by 0.76 m. Borders were removed by hand to an alley’s length of 60cm. Planting dates were 30 April 2010 and 24 April 2011. Plant stands were thinned to nine plants per square metre after establishment. Nitrogen applications were 20.2g m^–2^ and 13.5g m^–2^ of N in 2010 and 2011, respectively. Weeds were effectively controlled using 2-chloro-4-ethylamino-6-isopropylamino-*s*-triazine and *S*-metolachlor; mites and insects were effectively controlled using 2-oxo-3-(2,4,6-trimethylphenyl)-1-oxaspiro[4.4]non-3-en-4-yl 3,3-dimethylbutanoate and 3-Bromo-*N*-[4-chloro-2-methyl-6-[(methylamino)carbonyl]phenyl]-1-(3-chloro-2-pyridinyl)-1H-pyrazole-5-carboxamide.

Weather variables were from the Esparto weather station located near the experiment site and maintained by the California irrigation management information system (www.cimis.water.ca.gov/WSNReportCriteria.aspx). Cumulative precipitation between April and September was 97.1mm and 67.3mm in 2010 and 2011, respectively (Table 2), whereas total potential evapotranspiration for the same period was 1069mm in 2010 and 984mm in 2011. Supplemental irrigation was applied through a buried drip tape to promote good stand establishment, incorporation of fertilizer, and to mitigate heat stress at flowering time (Table 2). Temperatures greater than 40 °C during one month bracketing flowering were seven in 2010 and zero in 2011.

**Table 2.** Environment summary for 2010 and 2011 growing seasons Data are from the Esparto weather station, maintained by the California irrigation management and information system, located near the experiment site.

**Month**	**Mean solar** **radiation (W m** ^**–2**^)		**Mean temperature (° C**)		**Rainfall (mm**)		**Irrigation (mm**)	
	**2010**	**2011**	**2010**	**2011**	**2010**	**2011**	**2010**	**2011**
April	242	284	14.3	15.7	72.3	2.4		25.4
May	293	312	17.9	17.3	24.3	27.8	12.7	25.5
June	346	300	24.1	22.2	0.5	34.8	12.7	12.7
July	364	312	25.4	25.6	0	1.4	25.4	44.5
August	342	259	24.1	25.0	0	0		12.7
September	275	209	24.3	25.8	0	0.9		

### Phenotypic measurements

Phenotypic data on silk emergence and pollen shed were collected daily for a period of 3 weeks. Silking and shedding dates were determined when 50% of the plants in a plot exhibited at least one visible silk, or were shedding pollen, respectively. Thermal times to dates of silking and shedding were calculated using daily average temperature, a base temperature of 10 °C, and optimum temperature of 30 °C (Gilmore and Rogers, 1958). ASI was calculated as the difference between the thermal time to shedding and silking. Grain yield was measured using a New Holland automated research plot combine TR series (CNH Global, Burr Ridge, IL, USA) and adjusted to 0.15g g^–1^ moisture.

Soil moisture measurements were collected using a Time Domain Reflectometer (TDR) sensor model Trime T3-50 TAP (IMKO, Ettlingen, Germany). The instrument was calibrated by IMKO and corrected by bulk density measured in the fields (1.37–1.58g cm^–3^). The accuracy of the TDR instrument used in this study was 0.02cm^3^ cm^–3^ for an area of measurement of 180×150mm. One 3 m long access tube per plot was installed between the rows in the centre of each two-row plot when plants reached the two expanded leaves stage (V2). Images of root distribution profiles from excavations conducted to a depth of 1.7 m at physiological maturity and to a depth of 2.0 m at flowering time in experiments conducted at two locations—Viluco, Chile and Woodland, CA, USA, respectively—indicated that roots for a given genotype did not cross between two-row plots (images not shown). This observed root distribution pattern limits the plausible intermingling of roots close to the access tube, which is placed 1.14 m from the nearest planting row for a different genotype. Similar root architecture in maize, with nodal roots growing at an angle and then turning vertical, was observed in other studies (Tardieu and Pellerin, 1990; Ješko, 1992). Plant stands were uniform and no gaps were observed near the access tubes.

Table 1 indicates the hybrids for which soil moisture was measured. Fifteen measurements per tube were taken every 20cm between 23 June and 20 September in 2010, and from 2 May to 18 August in 2011. Data were collected in 11 d in 2010 and in 10 d in 2011. Water use at each layer was calculated as the difference between the first and the last measurement. Total water use was calculated by integrating soil water use across the soil column and adding irrigation amounts applied during the period of measurement. Rainfall amounts during this period (Table 2) had limited effect on total water use.

### Statistical analysis

The experiment was conducted as a randomized incomplete block design, with the factor hybrid randomized within replications, which were blocked to account for field variability. Experiments included four replications in 2010 and three replications in 2011. Formally, the data for flowering, yield, and ASI (*Y*_ijk_) of year (*E*)_i_, hybrid (*H*)_j_, block (*B*)_k_ within year, were modelled as a function of an overall mean *u*, factors for year, hybrid, and two-way way interaction between hybrid and year, block within year and the residual *e*_ijk_,

$$Y_{ijk}=u\;+\;E_{i}+\underline{H_{j}}+\underline{{\left(E\times H\right)}_{ij}}+\underline{{\left(B/E\right)}_{ik}}+\;\underline{e_{ijk}}$$

where random effects are denoted with underbars and fixed effects without underbars. Data for soil moisture (*Y*_*ijklm*_) of depth (*D*)_i_, year (*E*)_j_, date (*G*)_k_, hybrid (*H*)_l_, and block (*B*)_m_ were modelled as a function of an overall mean *u*, factors for depth, year, date, hybrid, block within year, two-way interactions depth by year, depth by date, depth by hybrid, year by hybrid, date by hybrid, depth by block within year, hybrid by block within year, three-way interactions depth by year by hybrid, depth by date by hybrid, and depth by hybrid by block within year; and a residual *e*_*ijklm*_,

$$\begin{array}{l} Y_{ijklm}=u\;+\;D_{i}+\underline{E_{j}}+\underline{G_{k}}+\underline{H_{l}}+\underline{{\left(B/E\right)}_{jm}+\;{\left(D\times E\right)}_{ij}}\\ \;+\underline{{\left(D\times G\right)}_{ik}}+\underline{{\left(D\times H\right)}_{il}}\\ \;+\underline{{\left(E\times H\right)}_{jl}}+\underline{{\left(G\times H\right)}_{kl}}\;\\ \;+\underline{{\left(D\times B/E\right)}_{ijm}}+\underline{{\left(H\times B/E\right)}_{jlm}}\\ \;+\underline{{\left(D\times E\times H\right)}_{ijl}}+\underline{{\left(D\times G\times H\right)}_{ikl}}\;\\ \;+\underline{{\left(D\times H\times B/E\right)}_{ijlm}}+\underline{e_{ijklm}} \end{array}$$

where random effects are denoted with underbars and fixed effects without underbars. Variance components of random effects were estimated by residual maximum likelihood method. An *F* test was used to assess significance for fixed effects. Analyses were conducted with ASREML (Gilmour *et al.*, 2009). Trends over year of release were tested for significance by linear regression (R Core Team, 2014).

## Results

### Phenology, yield, and ASI

Significant differences among hybrids in thermal time to shedding were observed in both 2010 and 2011 experiments (Table 3). However, trends for thermal time to shedding with respect to year of commercialization were not significant with regression slope [standard error (SE)] values –1.5 (±0.89) and –0.76 (±0.75) °C year^–1^ in 2010 and 2011, respectively.

**Table 3.** Variance components and SE for hybrid, experiment year, and interactions on grain yield, ASI, and time to shedding

**Source**	**Yield**		**ASI**		**Shedding**	
	**Component**	**SE**	**Component**	**SE**	**Component**	**SE**
Hybrid	7789*	5193	2051*	892	29.4*	11.3
Year × hybrid	7213*	4243	2590	3238	4.3*	2.5
Block year 1	3835	3835	0		0.8	0.8
Block year 2	2894	4134	0		0.6	1
Residual	13446*	2241	1096*	186	3.8*	0.7

Experiment year, hybrid, and block were included in the model as random sources of variation.

* Indicates variance components that are equal to or greater than 1.5 times their SEs.

Drought stress treatments were effective in reducing yields in both the 2010 and 2011 field experiments. Average yield in the experiment conducted in 2011 was significantly higher than that observed in 2010 (677 vs 317g m^–2^; *P*<0.001), and both markedly lower than yields close to 1400g m^–2^ reported for favourable conditions at this location (Cooper *et al.*, 2014). Average ASI was 154 °C and 175 °C for experiments conducted in 2011 and 2010, respectively. These positive values for ASI indicate that drought treatments affected reproductive development around flowering time and further demonstrate effective imposition of drought in these experiments.

**Fig. 1.**
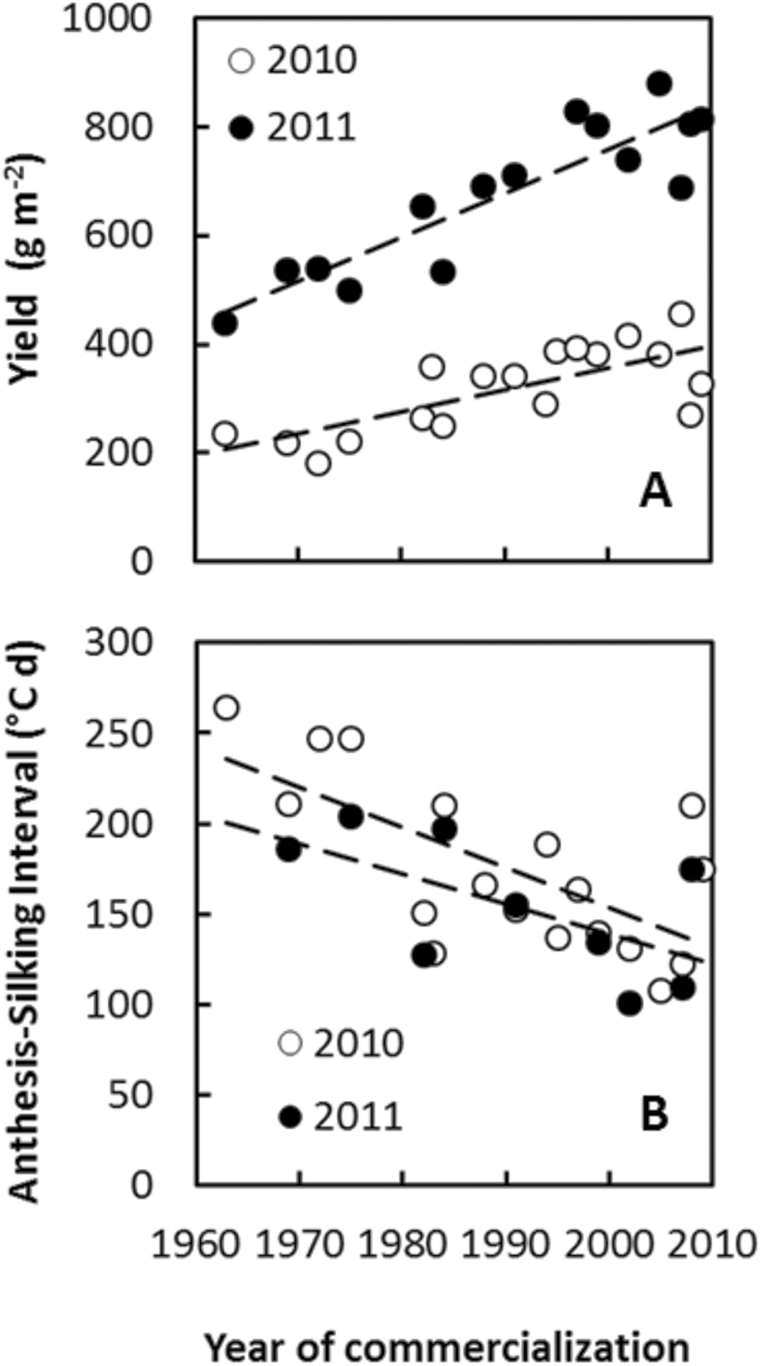
Best Linear Unbiased Predictions for yield (A) and ASI (B) for 2010 and 2011 experiments for hybrids commercialized between 1963 and 2009. The SE of the difference between hybrids is 62 for yield and 22.8 for ASI. Dotted lines represent least square regression lines. Trends with respect to year of commercialization for yield were 4.1(±0.95) (*P*<0.001) g m^–2^ year^–1^ for 2010 and 8.1(±1.1) (*P*<0.001) g m^–2^ year^–1^ for 2011. Trends for ASI were –2.2(±0.6) (*P*<0.01) and –1.7(±0.8) (*P*=0.078) °C year^–1^ for ASI in 2010 and 2011, respectively.

Hybrid variation was observed for both yield and ASI in both experiments (Table 3) and this was associated with year of commercialization (Fig. 1). In agreement with prior studies, yield under stress increased with later year of commercialization, and ASI showed the opposite pattern (Bolaños and Edmeades, 1993, 1996; Campos *et al.*, 2006). Yield under stress doubled between hybrids commercialized in 1963 and 2009. Estimated trends with respect to year of commercialization were 4.1(±0.95) (*P*<0.001) and 8.1(±1.1) (*P*<0.001) g m^–2^ year^–1^ for yield in 2010 and 2011, respectively, and –2.2(±0.6) (*P*<0.01) and –1.7(±0.8) (*P*=0.078) °C year^–1^ for ASI in 2010 and 2011, respectively. Because the high plant population may have been supra-optimal for the first cohort of SX hybrids, the trends in ASI may have been overestimated.

### Temporal patterns of water extraction

**Fig. 2.**
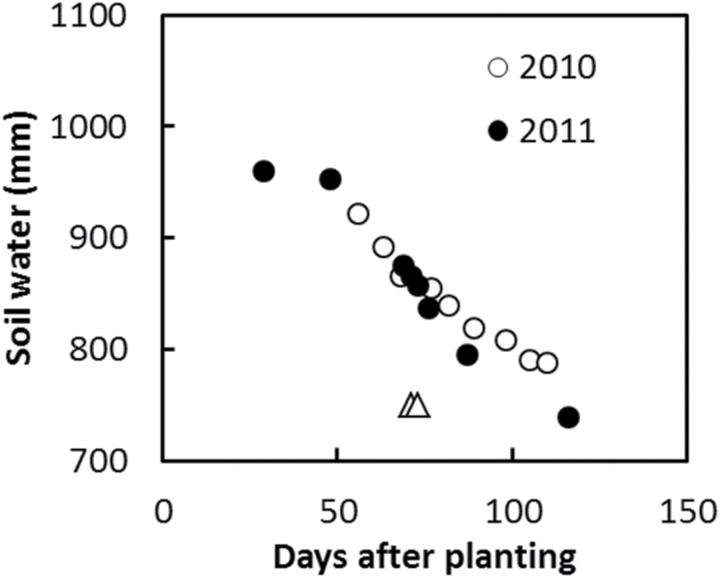
Best Linear Unbiased Predictions for total soil water content across hybrids for 2010 and 2011 experiments. Total soil water content is estimated by multiplying mean volumetric soil moisture and the soil depth (300cm). The SE of the difference between dates is 18.2mm. Flowering time, indicated by triangles, was 71 and 73 d after planting for 2010 and 2011, respectively.

The irrigation treatments resulted in a flowering stress followed by a grain filling and terminal stress during both years. In both experiments, soil moisture started to decline around 50 d after planting (Fig. 2), which was around 26 d prior to anthesis. Soil water declined gradually until maturity, when the crop was harvested (Fig. 2; Table 4).

**Table 4.** Variance components and SE for experiment year, date of measurement, hybrid, depth, block, and interactions on volumetric soil moisture

**Source**	**Component**	**SE**
Year	1.14	2.37
Block × year	0.09	0.11
Hybrid	0.14	0.13
Date	3.91*	1.48
Hybrid × year	0.00	-
Depth × year	0.00	0.12
Hybrid × block × year	0.53*	0.15
Depth × year × block	0.08*	0.05
Hybrid × depth	0.06	0.06
Hybrid × date	0.05*	0.01
Depth × date	2.19*	0.22
Depth × hybrid × year	0.00	-
Depth × hybrid × year × block	1.88*	0.11
Hybrid × depth × date	0.00	-
Error	0.91*	0.02

Experiment year, date of measurement, hybrid, and block were included in the model as random sources of variation.

* Indicates variance components that are equal to or greater than 1.5 times their SEs.

**Fig. 3.**
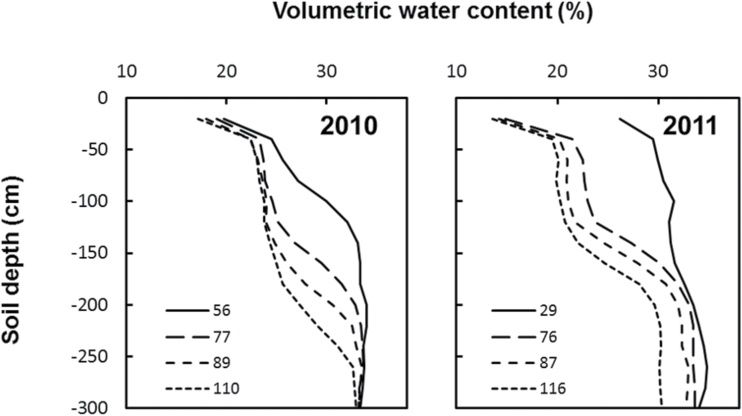
Best Linear Unbiased Predictions for soil water content across hybrids by soil depth for different days after planting for the 2010 and 2011 experiments. Lines indicate different measurement days. The SE of the difference between measurements is 0.75%.

As the growing season progressed, soil water was extracted from increasingly deeper soil layers (Fig. 3). The patterns of variation with date and depth were significantly different between years as indicated by the significant date by depth interaction variance component (Table 4). While in 2010 a clear soil water uptake front was observed progressing with depth across dates, in 2011 variation in soil water content was concentrated in the upper soil layers early in the growing season with a shift towards water use from the deeper soil layers post-flowering (Fig. 3). Significant soil water uptake was observed down to 2.4 m in 2010 and 3.0 m in 2011 (Fig. 3). These observations showed water uptake in soil layers deeper than prior reports for maize grown in the US Plains (Norwood, 2001; Payero *et al.*, 2006; Tolk *et al.*, 1998) and in an Argentinian silty loam Haplustoll (Dardanelli *et al.*, 1997). A significant variance component for hybrid by date interaction was observed for volumetric soil water content, but it was small relative to variance component for date (Table 4).

### Water uptake did not change during SX breeding

**Fig. 4.**
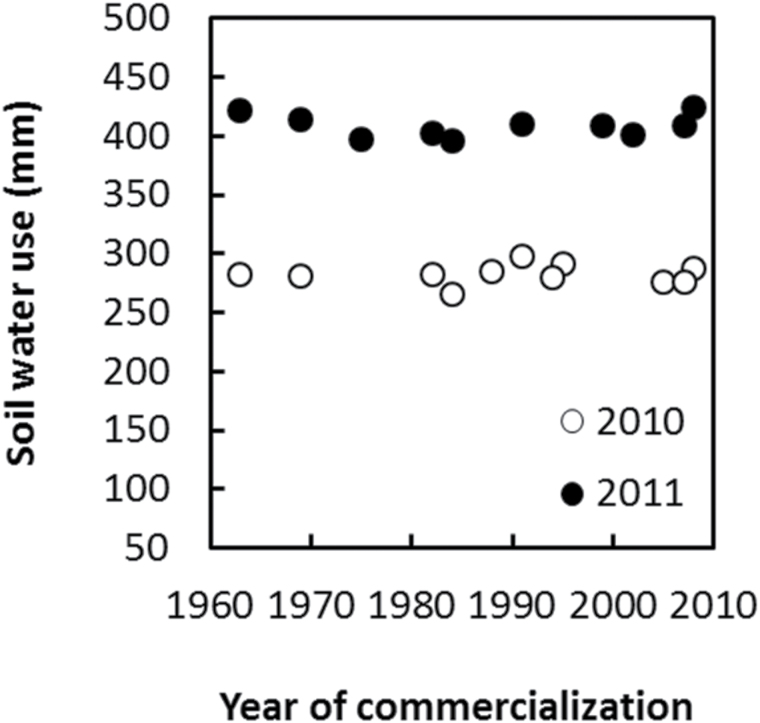
Soil water use of each hybrid over the measurement period versus year of hybrid commercialization for the 2010 and 2011 experiments. Soil water use was estimated as the difference between the Best Linear Unbiased Predictions for the first and last soil moisture measurements by hybrid and experiment year and including irrigation quantities. Trends with respect to year of commercialization for soil water use were 0.04(±0.19) (*P*=0.85) mm year^–1^ for 2010 and 0.016(±0.21) (*P*=0.94) mm year^–1^ for 2011.

Water uptake was estimated as the difference between the Best Linear Unbiased Predictions for soil moisture content at the first and the last measurements, with the addition of irrigation amounts. Figure 4 shows that total water use varied between years but not among hybrids. This result is consistent with the analysis of variance for soil moisture content (Table 4) that indicated absence or inability to detect a significant effect of hybrid on soil moisture content.

Total soil water use was greater in 2011 than in 2010 (Fig. 4), which was consistent with greater yields observed in 2011 relative to 2010 (Fig. 1). Variation between years in total soil water use was determined by differences in post-flowering water uptake (Fig. 2). At anthesis (approximately 71 and 73 d after planting in 2010 and 2011, respectively), water extraction was observed down to 1.8 m in 2010 and 1.6 m in 2011 (Fig. 3). Total soil moisture estimated at the time of anthesis was 862 and 857mm in 2010 and 2011, respectively. Higher irrigation applied in 2011 than in 2010 (Table 2) may have contributed to maintenance of leaf area, delayed senescence, and the observed higher post-flowering water use in 2011 than in 2010.

**Fig. 5.**
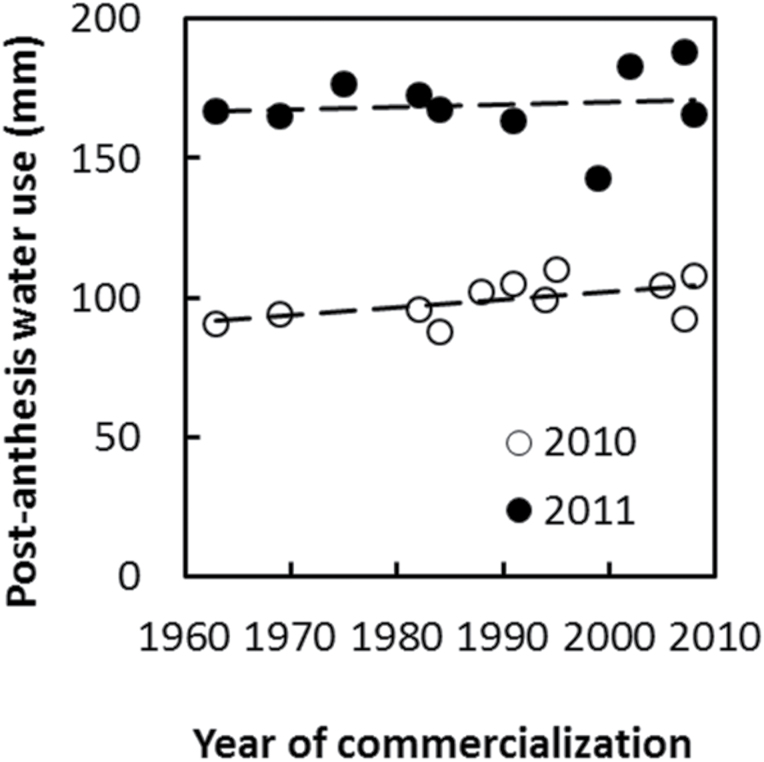
Soil water use post-anthesis of each hybrid versus year of hybrid commercialization for the 2010 and 2011 experiments. Soil water use was estimated as the difference between the Best Linear Unbiased Predictions for the last soil moisture measurement and the estimated soil moisture content at flowering time by hybrid and experiment year and including irrigation quantities. Soil moisture at anthesis for each hybrid was estimated by linear interpolation between the two Best Linear Unbiased Predictions for soil moisture bracketing the anthesis date. Trends with respect to year of commercialization for soil water use post-anthesis were 0.28 (±0.14) (*P*=0.078) mm year^–1^ for 2010 and 0.09(±0.27) (*P*=0.76) mm year^–1^ for 2011.

In addition to not differing in their total water use, hybrids did not differ significantly in post-anthesis water use (Fig. 5). Estimated trends with respect to year of commercialization were 0.28 (±0.14) (*P*=0.078) and 0.09 (±0.27) (*P*=0.76) mm year^–1^ for post-anthesis water use in 2010 and 2011, respectively This indicates that differences in timing of water use in relation to crop development were also not associated with year of commercialization during the period of SX breeding.

## Discussion

This study tested the hypothesis that yield improvement as evidenced within the ERA hybrid studies was caused at least in part by increased water uptake. The imposed levels of drought stress reduced yields to about 25% or less of yield attainable under favourable conditions. It also enabled measurement of soil water uptake in a terminal drought pattern that forced rooting to express the crop’s ability to extract water from deep soil layers (Tinker, 1976). The observed trends for the rate of gain in yield with year of commercialization (4–8g m^–2^ year^–1^) compared well with prior estimates for comparable yield levels and drought stress treatment (Campos *et al.*, 2006; Cooper *et al.*, 2014). A negative correlation between ASI and yield was also demonstrated as well as an overall positive ASI, which indicated that drought treatments affected reproductive development around flowering time (Bolaños and Edmeades, 1993, 1996; Edmeades *et al.*, 1993; Campos *et al.*, 2004).

Soil water extraction, however, remained constant across hybrids with no indication of changes related to year of hybrid commercialization. This fundamental result leads to the conclusion that over the period of SX breeding, total water uptake cannot explain the observed yield increase. Results do not rule out, however, that changes in water uptake could have occurred prior to the SX breeding era. The results therefore imply that the physiological underpinning of genetic improvement of yield during this period of SX breeding could be related to an increased allocation of biomass to the ear (Lafitte and Edmeades, 1995), perhaps at the expense of partitioning of biomass to root systems with the consequential improvement in root system efficiency (Bolaños *et al.*, 1993), maintenance of silk water status (Schoper *et al.*, 1987), sugar metabolism and flux (McLaughlin and Boyer, 2004), or efficiency of conversion of resources (Passioura, 1983).

### Constant water capture over the period of SX breeding

A fundamental result in this study is the demonstration that total soil water capture remained constant during the ~50 year period of SX breeding and was unrelated to year of hybrid commercialization. This result for US temperate maize complements prior findings for tropical (Bolaños *et al.*, 1993) and Argentinian germplasm (Nagore *et al.*, 2014). No differences were detected among eight cycles of selection of ‘Tuxpeño Sequia’ in seasonal profiles of water content down to 1.4 m (Bolaños *et al.*, 1993). Similarly, water capture did not differ among three SX Argentinian hybrids commercialized between 1980 and 2004, which was evident from non-significant differences in seasonal evapotranspiration, water use efficiency, and biomass production (Nagore *et al.*, 2014). In this last study, significant differences in soil moisture content were detected around flowering time. Genotypic variation in pattern of water use in maize was also shown for temperate maize (Cooper *et al.*, 2014). Similarly, a significant hybrid by date interaction was observed in the present study (Table 4). However, the magnitude of the variance component for date by hybrid was two orders of magnitude lower than the variance component for date suggesting that differences in the pattern of water use has not played a determinant role on pattern of yield gain as measured by association with year of commercialization. In addition, estimated trends in post-anthesis and total water use with respect to year of commercialization were not significant. The combined results from these studies thus provide strong evidence to reject the hypothesis that the observed long-term yield improvements achieved over the period of SX breeding were driven by increased water capture or the temporal pattern of water use through the crop life cycle.

The hypothesis postulated by Hammer *et al.* (2009) may still explain a component of the observed yield gain within the period of double-cross breeding. Between 1930 and 1970 there was a significant shift in root system architecture as inferred from root stability scores (Duvick *et al.*, 2004). A negative association between root mass and pulling resistance (Bolaños *et al.*, 1993) suggests that root stability, mass, and water capture may have all increased during this double-cross era of breeding.

### Water productivity increased since 1960

The corollary to the lack of association between yield improvement and water use is that genetic improvement for maize yield in the US corn-belt increased water productivity for yield between 0.018 and 0.013g mm^–1^ year^–1^. A simple identity (Passioura, 1983),

Yield = Water use × Transpiration efficiency × Harvest index,

can be used to estimate grain yield. In the absence of changes in water use with year of commercialization, and assuming changes in transpiration efficiency within the set of ERA hybrids included in this study were negligible—which is supported by experimental evidence from studies on Argentinean hybrids (Nagore *et al.*, 2014) and sweet corn varieties (Bunce, 2011)—the observed variation in yield must be related to changes in HI under stress. An increase in HI could be due to effects of selection on reproductive resilience and biomass allocation to the ear at expense of root systems, similar to observed changes in tropical maize (Bolaños and Edmeades, 1993; Bolaños *et al.*, 1993; Lafitte and Edmeades, 1995) and Argentinian semi-flints (Echarte *et al.*, 2000).

### Implications for genetic improvement

Breeding trajectories for complex traits in rugged yield-trait landscapes are anything but simple (Hammer *et al.*, 2006; Messina *et al.*, 2011). Trade-offs among adaptive traits, frequency of environment types, structure of breeding programs, and access to genetic diversity among other factors, determine the direction and rate of observed change in genetic gain. Long-term simulation studies demonstrate a diversity of plausible breeding trajectories for a given environment-crop-breeding system with physiological traits changing in overlapping sequences and in a non-linear manner (Messina *et al.*, 2011). When placed in the context of long-term selection (Duvick *et al.*, 2004), this study focused on a relatively short time span of breeding. As such, and in the context of rugged yield-trait landscapes, it is conceivable that current observations on water capture showed a transient plateau. Prior to SX breeding, selection for improved yield stability and reduced root lodging (Cooper *et al.*, 2006) could have increased the ability of maize to capture water resources, as suggested by the hybrid water use results reported by Campos *et al.* (2004). During SX breeding, selection operated to realize an intrinsic water capture and yield potential attained during double-cross breeding but limited by the susceptibility of maize reproductive physiology to stress. Further increase in this potential will have to rely on improvements in either resource use efficiencies or in water capture. Thus, it is opportune to evaluate the feasibility to design root systems with improved water capture beyond current levels, which can improve plant water status in the 3 weeks around flowering time. Simulation studies for drought prone environments and for the central US corn-belt indicate that changes in root occupancy can increase yields (Hammer *et al.*, 2009; Messina *et al.*, 2009, 2011). However, meaningful phenotyping of root systems architecture is poorly defined in maize. Despite changes in root architecture in the last 100 years (York *et al.*, 2015), the results of the present study indicated that potential water capture remained constant over the period of breeding represented by the sequence of hybrids, which questions the value of these traits as predictors of increasing water resource capture and their value to inform selection decisions. Modelling approaches are being evaluated to understand root form and function effects on whole plant water status (Draye *et al.*, 2010; Leitner *et al.*, 2014). Application of modelling and meaningful phenotyping proved useful to inform breeding approaches for sorghum and wheat in Australia (Manschadi *et al.*, 2006, 2008; Kirkegaard *et al.*, 2007; Singh *et al.*, 2012). It is anticipated that integration of modelling technologies, field, and controlled environment phenotyping, and genetic studies in maize will bring opportunities to develop improved and more stable germplasm that fully utilizes available soil water resources.
